# The Value of Fournier’s Gangrene Scoring Systems on Admission to Predict Mortality: A Systematic Review and Meta-Analysis

**DOI:** 10.3390/jpm13091283

**Published:** 2023-08-22

**Authors:** Antonio Tufano, Piervito Dipinto, Francesco Passaro, Umberto Anceschi, Giorgio Franco, Rocco Simone Flammia, Flavia Proietti, Luca Antonelli, Giovanni Battista Di Pierro, Francesco Prata, Roberta Rullo, Sisto Perdonà, Costantino Leonardo

**Affiliations:** 1 Department of Maternal-Infant and Urological Sciences, “Sapienza” Rome University, Policlinico Umberto I Hospital, 00162 Rome, Italy; pierdip97@gmail.com (P.D.); giorgio.franco@uniroma1.it (G.F.); roccosimone92@gmail.com (R.S.F.); flavia.proietti@uniroma1.it (F.P.); luca.anto.92@gmail.com (L.A.); giovannibattista.dipierro@uniroma1.it (G.B.D.P.); 2 Department of Neurosciences, Reproductive Sciences and Odontostomatology, University of Naples “Federico II”, 80131 Naples, Italy; francescopassaro1996@gmail.com; 3 Department of Urology, “Regina Elena” National Cancer Institute, 00128 Rome, Italy; umberto.anceschi@gmail.com (U.A.); costantino.leonardo@gmail.com (C.L.); 4 Department of Urology, Fondazione Policlinico Universitario Campus Bio-Medico, 00128 Rome, Italy; f.prata@unicampus.it; 5 Obstetrics and High-Risk Pregnancy Unit, Department of Woman, Child Health and Public Health, Fondazione Policlinico Universitario A. Gemelli IRCCS, Largo Agostino Gemelli 8, 00168 Rome, Italy; rulloroberta90@gmail.com; 6 Istituto Nazionale Tumori di Napoli, IRCCS “Fondazione G. Pascale”, Via M. Semmola, 80131 Naples, Italy; s.perdona@istitutotumori.na.it

**Keywords:** Fournier gangrene, mortality, infection, Fournier gangrene severity index, FGSI

## Abstract

Objective: To systematically review and meta-analyze the predictive value of the Fournier gangrene severity index (FGSI), the simplified FGSI (SFGSI), and the Uludag FGSI (UFGSI) on mortality in patients affected by Fournier’s Gangrene (FG). Methods: A search was performed in PubMed, Web of Science, Embase, and the Cochrane Library, from January 2000 to May 2023, to identify original cohorts comparing data between surviving and non-surviving FG patients. The statistical analysis consisted of two parts. First, the mean and standard deviation (SD) of the FGSI, SFGSI, and UFGSI at admission were extrapolated from each study, and the pooled mean difference (MD) with 95% confidence interval (95% CI) was obtained using the Der Simonian–Laird random-effect model. Second, to evaluate the accuracy of the FGSI, SFGSI, and UFSGI in predicting mortality, true positive (TP), false positive (FP), true negative (TN), and false negative (FN) values were extracted where possible and reported in 2 × 2 contingency tables. The sensitivity, specificity, and AUC values were pooled, and summary receiver operating characteristic (SROC) curves were constructed. Results: Overall, forty studies comprising 2257 patients were included. The pooled analysis revealed that the FGSI, SFGSI, and UFGSI values at admission were higher in non-survivors than survivors (MD: 5.53 (95% CI: 4.68–6.37); MD: 2.41 (95% CI: 1.06–3.77); and MD: 5.47 (95% CI: 3.68–7.26), respectively). Moreover, the AUC values of the FGSI, SFGSI, and UFGSI were 0.90 (95% CI: 0.87–0.92), 0.84 (95% CI: 0.80–0.87), and 0.94 (95% CI: 0.92–0.96), respectively. Conclusions: The higher scores of the FGSI, SFGSI, and UFGSI on admission were associated with mortality. Moreover, when comparing accuracy rates, the UFGSI exhibited the highest AUC value.

## 1. Introduction

Fournier’s gangrene (FG) is a rare and potentially life-threatening necrotizing fasciitis that affects the genital and perineal regions. FG infection is often polymicrobial, caused by a synergy of aerobic and anaerobic bacteria. Despite the substantial advancements in understanding the underlying causes and mechanisms of FG, the mortality rates related to this condition are still elevated, ranging from 5% to 65% [1,2]. 

To date, the clinical management of FG consists of extensive surgical debridement, fascia incision, the drainage of necrotic tissue, and sufficient intravenous administration of broad-spectrum antibiotics [3]. Moreover, recent studies have also highlighted the potential benefits of hyperbaric oxygen therapy [4]; however, its application is still limited, due to challenges in accessing and transferring patients to units that offer this service.

In the last decades, several scoring systems have been conceived to assess the severity of FG at the time of admission. The Fournier gangrene severity index (FGSI), developed by Laor et al. in 1995, represents one of the most commonly used scoring systems able to predict the likelihood of mortality in patients with FG [5]. More specifically, the FGSI incorporates nine clinical and laboratory parameters (including temperature, heart rate, respiratory rate, sodium, potassium, creatinine, leukocytes, hematocrit, and bicarbonate). In this context, each parameter is given a score on a scale from 0 to 4, and the overall score is calculated by adding together the points assigned to each parameter. Later, in 2010, Yilmazlar et al. proposed the Uludag FGSI (UFGSI) by incorporating the age and the extent of the disease to the FGSI [6]. Here, the authors found that the UFGSI outperformed the FGSI in predicting mortality (ROC curves: 0.94 vs. 0.84). Finally, a shorter version of the FGSI, known as the simplified FGSI (SFGSI), was introduced by Lin et al. by including only three variables (serum potassium, creatinine, and hematocrit), with the aim of establishing a more user-friendly score while at the same time maintaining a high sensitivity and specificity [7].

The aim of this systematic review and meta-analysis is to investigate the predictive values and accuracy of the FGSI, SFGSI, and UFGSI on mortality in patients affected by FG.

## 2. Materials and Methods

### 2.1. Study Design and Search Strategy

This meta-analysis was conducted in accordance with the preferred reporting items for systematic review and eta-analyses (PRISMA) guidelines [8]. A literature search using electronic databases (PubMed, Web of Science, Embase, and the Cochrane Library) was performed from January 2000 to May 2023. The search strategy included a comprehensive set of the following keywords: (“Fournier”[All Fields] OR “fourniers”[All Fields] OR “fourniers”[All Fields]) AND “FGSI”[All Fields]; (“Fournier gangrene”[MeSH Terms] OR (“Fournier”[All Fields] AND “gangrene”[All Fields]) OR “Fournier gangrene”[All Fields]) AND “SFGSI”[All Fields]; (“Fournier gangrene”[MeSH Terms] OR (“Fournier”[All Fields] AND “gangrene”[All Fields]) OR “Fournier gangrene”[All Fields]) AND “UFGSI”[All Fields]. Only English-language studies published in peer-reviewed journals were included.

### 2.2. Eligibility Criteria

All relevant original publications reporting FGSI, UFGSI and SFGSI scores at admission and comparing data between surviving and non-surviving FG patients were included.

The clinical and laboratory parameters constituting the FGSI, UFGSI and SFGSI are shown in Table 1.

Studies were excluded if they met one of the following exclusion criteria: (1) in vitro or animal study; (2) data duplication, data overlapping, or unreliably extracted or incomplete data; (3) abstract only article, review, thesis, book, conference paper, editorial, author response, letter, and comments; (4) article without available full text along with (5) any previous systematic review, meta-analysis, or literature review on the topic of interest. After deduplication, title screening and abstract screening were performed by two independent reviewers (A.T. and F.P.) to select the relevant studies. Eligible articles were further screened for inclusion in the systematic review and meta-analysis. Any disagreement was resolved by discussion and by consulting the senior reviewer when necessary (U.A.).

### 2.3. Data Extraction and Management

For each database, we downloaded all references that matched the final search terms using Endnote X9 software (Version 3.3, Clarivate Analytics, London, UK). Moreover, we examined the reference list of the eligible studies identified during the screening process to search for any additional studies. After the selection of articles based on the predefined inclusion criteria, the full texts were subject to data extraction by two authors using a Microsoft Excel file. Data were rechecked by at least two independent reviewers to ensure the accuracy of the extracted data. All disagreements and discrepancies were resolved by discussion and consultation with a senior team member when necessary (C.L.). Papers published by the same research group and studying the same factors were checked for potential duplicate data based on the year of patient recruitment and the hospital where the patients were recruited and by confirmation from the study authors. The study that had the longest follow-up time was selected for inclusion.

### 2.4. Quality Assessment

The quality of each study was independently evaluated by two investigators (P.D. and A.T.) according to the National Institutes of Health’s (NIH) quality assessment tool (Supplementary Table S1) [9]. The assessment of each study’s quality was achieved using a scoring system comprising 14 questions. The interpretation of the scores was as follows: studies obtaining a score of 13–14 were categorized as exhibiting good quality, while those scoring between 9 and 12 were deemed fair. Studies scoring below 9 were regarded as poor quality, specifically in the context of cohort studies. Divergences and discrepancies were resolved through discussion or consultation with a third party.

### 2.5. Statistical Analysis and Data Synthesis

The statistical analysis consisted of 2 parts. First, studies reporting the mean and standard deviation (SD) of the FGSI, SFGSI, and UFGSI at the time of admission between two cohorts (survivors vs. non-survivors) were extracted, and the pooled mean differences (MD) with 95% confidence interval (95% CI) were obtained using the Der Simonian–Laird random-effect model. To estimate the mean and standard deviation for non-normal data based on the provided median and interquartile range (IQR), we utilized the approach described by Hozo et al. [10]. The heterogeneity between studies was evaluated with *p* value and I^2^. I^2^ ≥ 50% or *p* ≤ 0.05 was deemed to represent significant heterogeneity. On the contrary, if statistical study heterogeneity was not observed (I^2^ ≤ 50% and *p* ≥ 0.05), a fixed-effects model was used. 

Second, to evaluate the accuracy of the FGSI, SFGSI, and UFSGI, true positive (TP), false positive (FP), true negative (TN), and false negative (FN) values were extracted, whenever possible, from each study and reported in 2 × 2 contingency tables. Finally, sensitivity, specificity, and AUC from different studies were pooled and summary receiver operating characteristic (SROC) curves were constructed. 

Publication bias was evaluated both qualitatively and quantitatively in this study. Qualitatively, the assessment was conducted using funnel plots by comparing the standardized mean difference (SMD) and the standard error of the natural logarithm of SMD (SE(SMD)) (Supplementary Figures S1–S3). Additionally, a quantitative analysis was performed using Galbraith plots to determine the presence of small-study effects (Supplementary Figures S4–S6). All of the analyses were accomplished using Stata version 18 (Stata Corporation, College Station, TX, USA), with all tests being two-sided, and with a statistical significance set at <0.05.

## 3. Results

### 3.1. Included Studies and Study Characteristics

**Figure 1:**
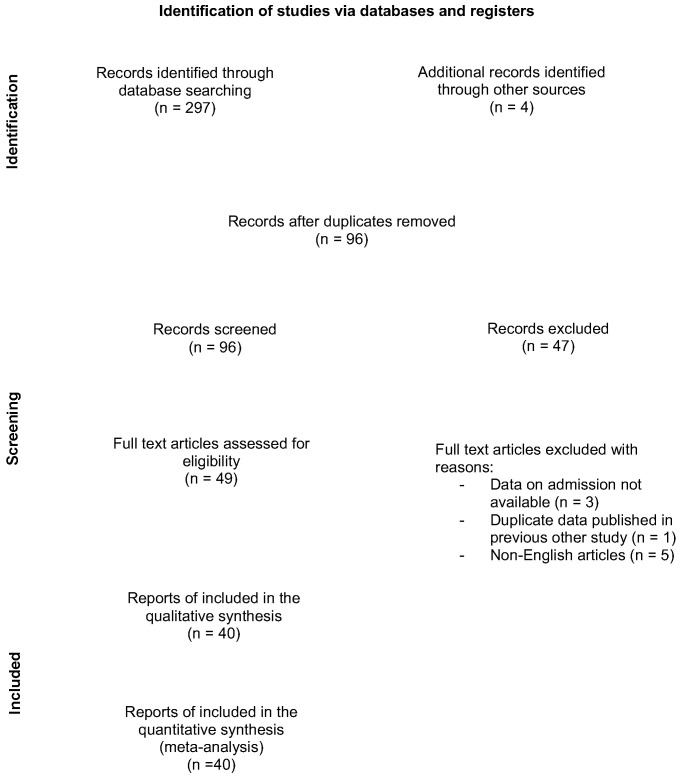
Flow diagram showing the number of abstracts and articles screened and evaluated during the review process.

Our search initially yielded 301 article references (Figure 1). Of those, 205 were subsequently removed, due to either duplication or not meeting the inclusion criteria. Full-text articles were then re-evaluated and critically analyzed for the remaining 49 references. Of those, nine were excluded, with reasons. Finally, the remaining 40 articles [6,7,11,12,13,14,15,16,17,18,19,20,21,22,23,24,25,26,27,28,29,30,31,32,33,34,35,36,37,38,39,40,41,42,43,44,45,46,47,48], comprising a total of 2257 patients, were considered for our systematic review and meta-analysis. The main characteristics of the included studies are detailed in Table 2. Of those, 36 were single-center studies and 4 were multi-center studies. Moreover, 38 studies were retrospective, and only 2 were prospective. The sample size varied between 16 and 150 patients, with a mean age ranging from 44.6 to 65.9 years.

### 3.2. The Prognostic Value of FGSI

**Figure 2:**
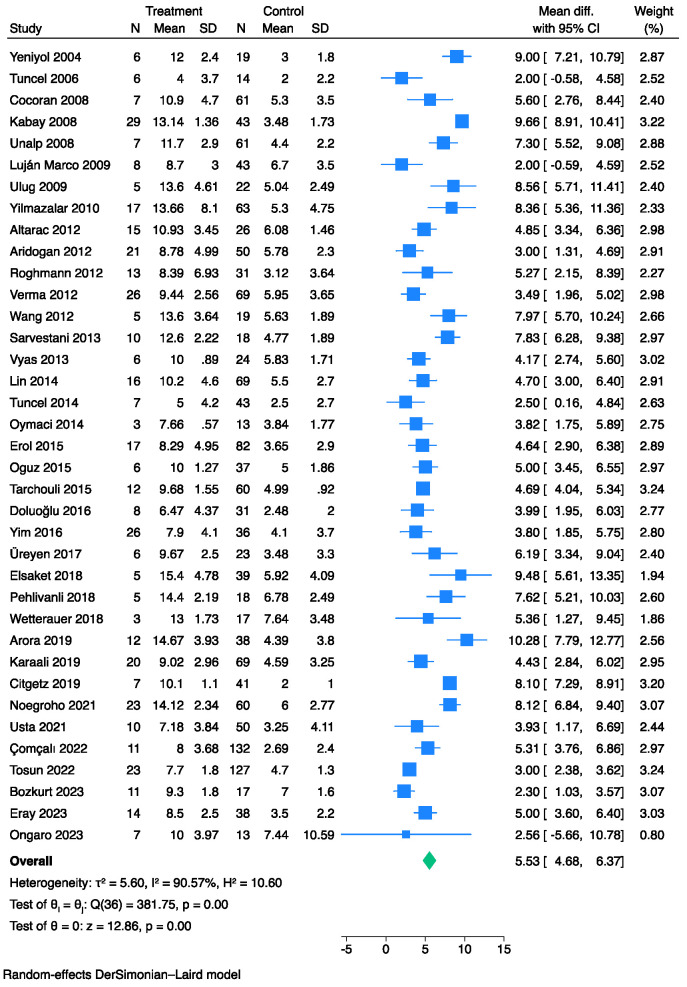
Forest plot for studies using the Der Simonian-Laird random-effect models showing elevated FGSI scores on admission in non-survivors vs. survivors.

Overall, 37 studies [6,7,11,12,13,14,15,16,17,18,19,20,21,22,23,24,25,26,27,28,29,30,31,32,33,34,36,37,38,39,41,42,43,44,45,47,48] comprising 2043 patients with FG and evaluable FGSI data at admission were compared between survivors and non-survivors. The pooled analysis showed that the non-survivor cohorts exhibited higher FGSI values than the survivors (MD: 5.53, 95% CI: 4.68–6.37) (Figure 2). However, significant heterogeneity was observed across the studies (I^2^: 91%). 

**Figure 3:**
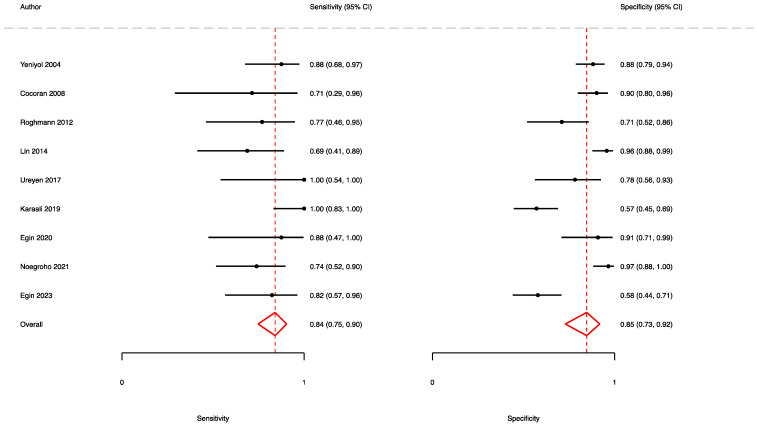
Forest plot showing the sensitivity and specificity of the FGSI in predicting mortality.

**Figure 4:**
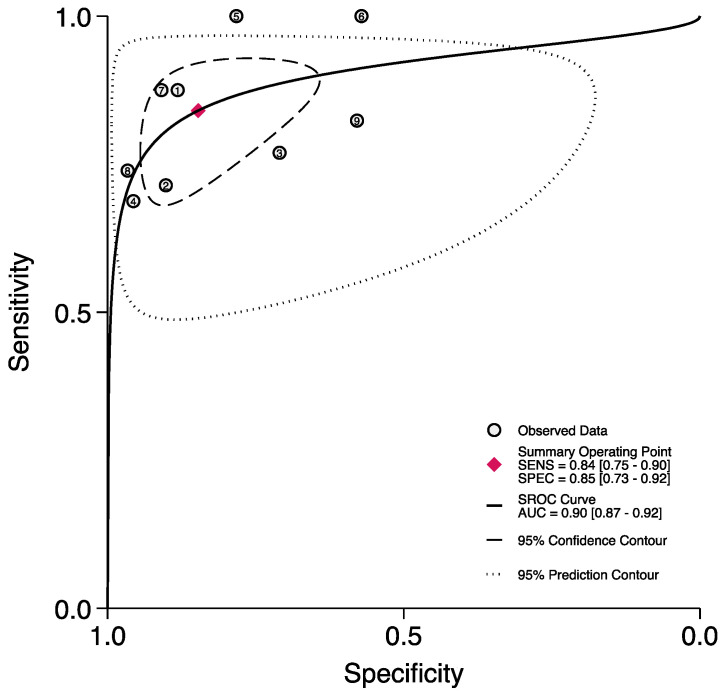
ROC curve and AUC of the FGSI in predicting mortality.

A total of nine studies [7,11,13,20,32,39,40,41,46] reported the accuracy rates of the FGSI in predicting mortality. Here, the pooled sensitivity, specificity, and AUC values were 0.84 (95% CI (0.75–0.90)), 0.85 (95% CI (0.73–0.92)), and 0.90 (95% CI (0.87–0.92)), respectively (Figure 3 and Figure 4).

### 3.3. The Prognostic Value of SFGSI

**Figure 5:**
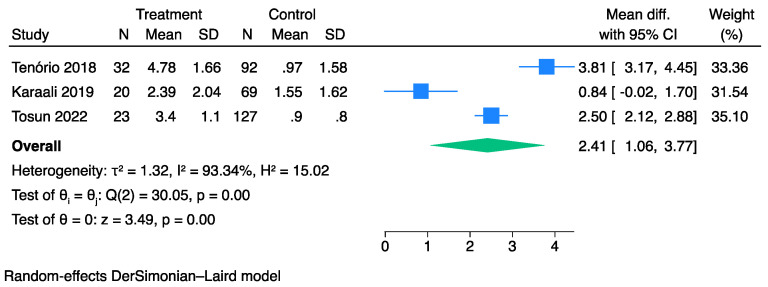
Forest plot for studies using the Der Simonian-Laird random-effect models showing elevated SFGSI scores on admission in non-survivors vs. survivors.

Overall, three studies [35,39,44] comprising 363 patients with FG and evaluable SFGSI data at admission were compared between survivors and non-survivors. The pooled analysis showed that the non-survivor cohorts exhibited higher SFGSI values than the survivors (MD: 2.41, 95% CI: 1.06–3.77) (Figure 5). However, significant heterogeneity was observed across the studies (I^2^: 93%,). 

**Figure 6:**
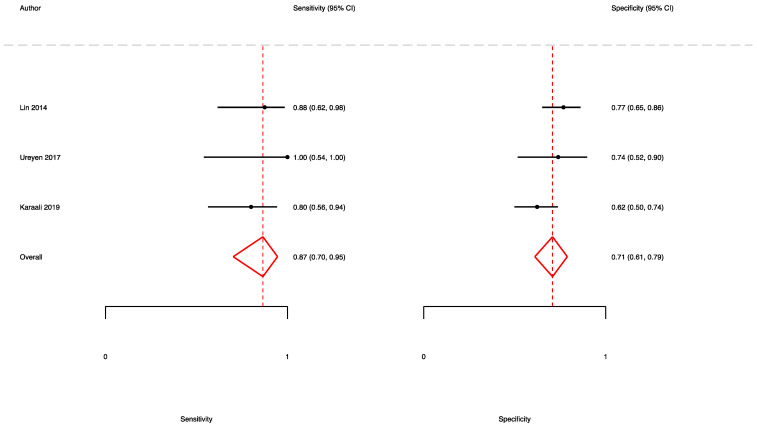
Forest plot showing the sensitivity and specificity of the SFGSI in predicting mortality.

**Figure 7:**
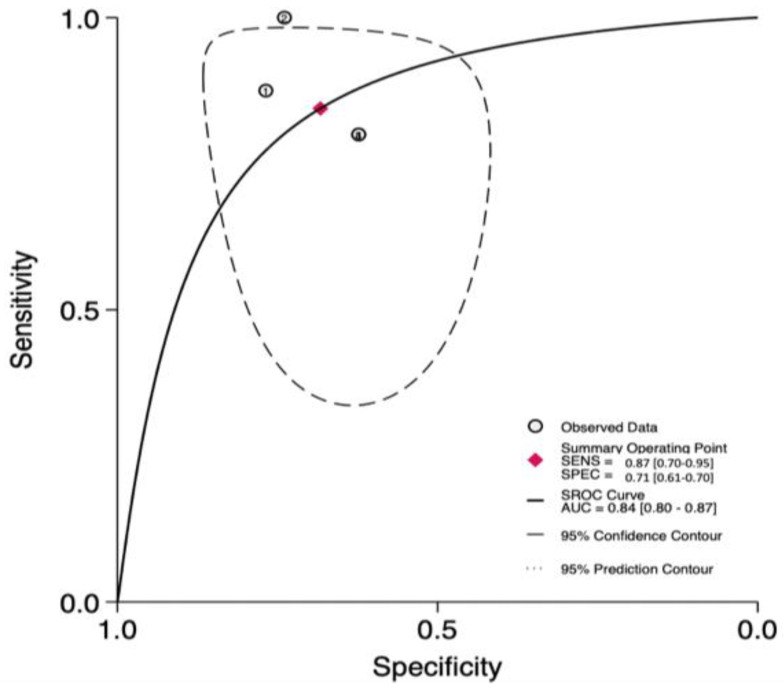
ROC curve and AUC of the SFGSI in predicting mortality.

A total of three studies [7,32,39] reported the accuracy rates of the SFGSI in predicting mortality. Here, the pooled sensitivity, specificity, and AUC values were 0.87 (95% CI (0.70–0.95)), 0.71 (95% CI (0.61–0.79)), and 0.84 (95% CI (0.80–0.87)), respectively (Figure 6 and Figure 7). 

### 3.4. The Prognostic Value of UFGSI

**Figure 8:**
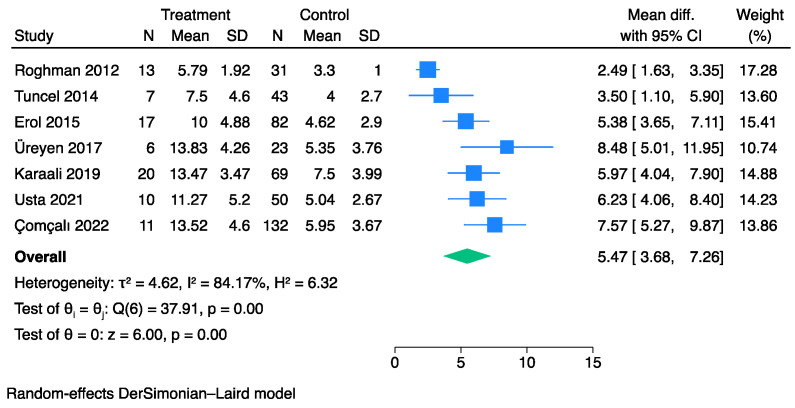
Forest plot for studies using the Der Simonian–Laird random-effect models showing elevated UFGSI scores on admission in non-survivors vs. survivors.

Overall, seven studies [20,26,27,32,39,42,43] comprising 515 patients with FG and evaluable UFGSI data at admission were compared between survivors and non-survivors. The pooled analysis showed that the non-survivor cohorts exhibited higher UFGSI values than the survivors (MD: 5.47, 95% CI: 3.68–7.26) (Figure 8). However, significant heterogeneity was observed across the studies (I^2^: 84%). 

**Figure 9:**
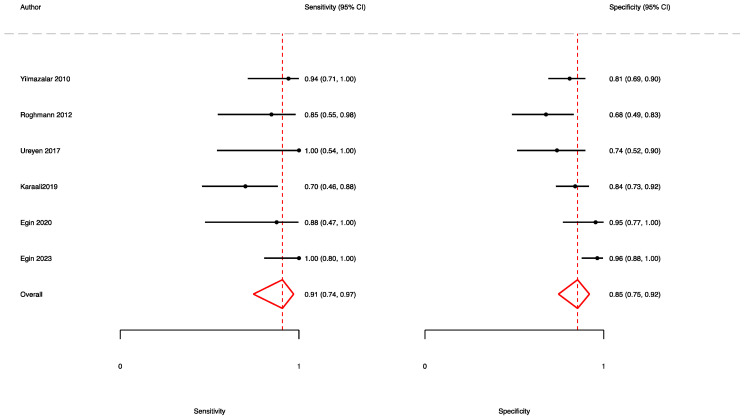
Forest plot showing the sensitivity and specificity of the UFGSI in predicting mortality.

**Figure 10:**
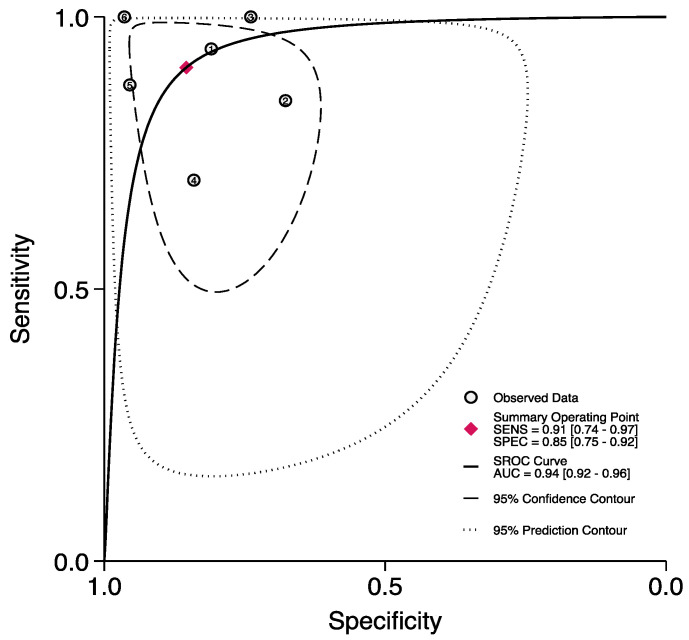
ROC curve and AUC of the UFGSI in predicting mortality.

A total of six studies [11,20,32,39,40,46] reported the accuracy rates of the FGSI in predicting mortality. Here, the pooled sensitivity, specificity, and AUC values were 0.91 (95% CI (0.74–0.97)), 0.85 (95% CI (0.75–0.92)), and 0.94 (95% CI (0.92–0.96)), respectively (Figure 9 and Figure 10).

## 4. Discussion

FG is usually a polymicrobial infection caused by aerobic Gram-negative bacilli or Gram-positive cocci. The most frequently identified bacterial species, upon culture, are Enterobacteriaceae, such as *E. Coli*, followed by streptococcal species [49]. Other organisms that are frequently isolated include *Staphylococci*, *P. aeruginosa*, *Peptostreptococci*, *Bacteroides* spp., and *Clostridia* [49].

Several comorbidities, such as diabetes, heart disease, renal failure, and kidney disease, may represent significant factors of increased mortality rates in individuals with FG [50]. Nevertheless, abnormal laboratory parameters at admission are recognized as important predictors of survival outcomes [5,6,7]. More specifically, increased leukocyte counts and levels of creatinine, creatine kinase, urea, lactate dehydrogenase, alkaline phosphatase, and decreased levels of hematocrit, bicarbonate, sodium, potassium, calcium, total protein, and albumin, are most often predictive of a worse prognosis [5,6,7,50]. Relying on these parameters, the FGSI, SFGSI, and UFGSI are commonly used scoring systems in evaluating the severity of FG patients at the time of admission.

The results of this meta-analysis suggest that patients affected by FG present higher scores of the FGSI, SFGSI, and UFGSI at admission. Our analyses led to several noteworthy findings. First, the FSGI values were higher in non-survivors than in survivors (MD: 5.53, 95% CI: 4.68–6.37). Hence, these laboratory abnormalities likely indicate sepsis associated with acute kidney injury and could represent the initial phase of multi-organ failure. Moreover, we also focused on studies estimating the accuracy of the mortality prediction of the FGSI [7,11,13,20,32,39,40,41,46]. Here, the sensitivity of the FGSI ranged from 69% to 100%, and the specificity ranged from 57% to 97% [7,32,39,41], depicting a pooled AUC value of 0.90 (95% CI: 0.87–0.92). Although no consensus has yet been established in determining an optimal cut-off value for the FGSI, a threshold of nine was most commonly retrieved among the included articles. However, we are aware that heterogeneity of the cut-off value used may lead to inevitable potential bias.

Second, the UFGSI was developed by Yilmazlar et al. in 2010 by incorporating the age and the extent of disease to the FGSI parameters [6]. In our meta-analysis, Çomçalı et al. represented the largest cohort (n = 144) evaluating the UFGSI as a predictor of mortality in FG patients, showing an AUC value of 0.89 [43]. Notably, the authors also proposed a novel score, named the Fournier’s gangrene mortality estimation model (FGMPM) score, which incorporates some parameters from the FGSI, UFGSI, and age-adjusted Charlson comorbidity index (ACCI) scores, with variables such as the depth of necrosis, the need for intensive care, the requirement for inotropes, and the neutrophil–lymphocyte ratio. Interestingly, the FGMPM depicted the highest AUC value among all of the evaluated scores (0.985; 95% CI: 0.998–1.000) [43]. However, despite the strength of the FGMPM, future studies are needed to externally validate this index as a reliable predictive scoring system. Nevertheless, the authors found that the UFGSI outperformed the FGSI, as evidenced by a statistically significative difference between the two ROC curves (0.105) (*p* = 0.002) [6]. This is in agreement with the results of our meta-analysis, where the pooled AUC in predicting mortality of the UFGSI was higher when compared to the FGSI and SFSGI (0.94 vs. 0.90 vs. 0.84). 

The SFGSI was developed by Lin et al., relying on the theory that most of the current studies indicated a consistent trend of elevated serum creatinine levels or the presence of renal failure as cornerstones of a poorer prognosis in patients with FG [7]. The authors found a noteworthy relationship between patient mortality and levels of serum creatinine, potassium, and hematocrit. Notably, these three parameters are frequently observed to be abnormal in patients with renal failure, suggesting that renal function might play a crucial role in influencing the overall outcome of individuals with FG [51,52,53]. This association was further reinforced by a large population-based cohort study (n = 1641), where renal failure emerged as a significant predictor of mortality, with an odds ratio of 5.3 [54]. Tenorio et al. confirmed these variables to be independent risk factors of mortality with a univariate analysis [35]. Additionally, when tested with a multivariate analysis, a significant increase in odds ratio was depicted (O.R.:50.2; 95% CI: 13.18–191.47), and patients with an SFGSI score greater than two experienced a mortality rate of 70%, whereas patients with an SFGSI score lower than two had a significantly lower mortality rate of 4.8% (*p* < 0.0001) [35]. Based on our results, we found a higher SFGSI MD between non-survivors and survivors (MD: 2.41, 95% CI: 1.06–3.77), with a pooled AUC value of 0.84 (95% CI 0.80–0.87), in predicting mortality. Finally, the SFGSI appears to be a promising alternative for assessing the mortality predictors in Fournier’s gangrene. Its main advantage lies in its straightforward applicability, as it involves only three parameters. Furthermore, it can be readily employed right after the patient’s admission, allowing for early risk assessment and timely intervention. 

Taken together, the FGSI, UFGSI and SFGSI are extremely useful tools for clinicians in evaluating patients with FG in daily clinical practice. However, the time-consuming nature of the FGSI and UFGSI scoring systems has led to the development of new and more practical scoring systems, such as NLR, PLR, and RDW [55]. However, these systemic inflammatory response markers have not been thoroughly evaluated, and the absence of well-established cut-off values, as well as the low number of studies, did not allow us to quantitatively pool these systemic inflammatory response markers. 

The major strength of the current study is the high number of studies included (n = 40), enrolling a total of 2257 patients. To the best of our knowledge, this is the first meta-analysis investigating the prognostic values of the FGSI, SFGSI, and UFGSI in FG patients.

We are aware of the limitations of this meta-analysis. First, although the association between the FGSI/UFGSI and mortality risk in FG was clear, we failed to find a uniformed cut-off value. Second, the mortality rates varied among the studies, which may be attributed to the variations in the baseline characteristics of the enrolled patients in each study, resulting in heterogeneity in our reported outcomes. Pre-existent comorbidities, the duration of symptoms, surgical delay, and poor renal function may represent important confounders. Third, the included studies were more frequently retrospective; therefore, selection bias, recall bias, and other biases should be considered, which may also cause heterogeneity in the pooled outcomes. Finally, the large ethnic diversity and small sample size of some studies may have caused sampling error [56]. Further studies with larger populations and a sufficient patient number are required to validate our study results.

## 5. Conclusions

In our meta-analysis, higher scores of the FGSI, SFGSI, and UFGSI on admission were associated with mortality. Moreover, when comparing the AUC values, the UFGSI exhibited the highest accuracy, followed by the FGSI. Future studies are needed to externally validate the new scoring systems.

## Figures and Tables

**Table 1:** Clinical and laboratory parameters constituting the three evaluated scoring systems (FGSI, UFGSI, and SFGSI).

FGSI			High		Normal		Low		
	**+4**	**+3**	**+2**	**+1**	**0**	**+1**	**+2**	**+3**	**+4**
*Temp.* °C	>41	39–40.9	-	38.5–38.9	36–38.4	34–35.9	32–33.9	30–31.9	<39.9
*Heart rate*	>180	140–179	110–139	-	70–109	-	55–69	40–54	<39
*Respiratory rate*	>50	35–49	-	25–34	12–24	10–11	6–9	-	<5
*Serum sodium* mmol/L	>180	160–179	155–159	150–154	130–149	-	120–129	111–119	<110
*Serum potassium* mmol/L	>7	6–6.9	-	5.5–5.9	3.5–5.4	3–3.4	2.5–2.9	-	<2.5
*Serum creatinine* mg/100 mL	>3.5	2–3.4	1.5–1.9	-	0.6–1.4	-	<0.6	-	-
*Hematocrit (HT)*	>60	-	50–59.9	46–49.9	30–45.9	-	20–29.9	-	<20
*Leukocytes total*/mm^3^ × 1000	>40	-	20–39.9	15–19.9	3–14.9	-	1–2.9	-	<1
*Serum bicarbonate* mmol/L	>52	41–51.9	-	32–40.9	22–31.9	-	18–21.9	15–17.9	<15
**UFGSI**			High		Normal		Low		
	**+4**	**+3**	**+2**	**+1**	**0**	**+1**	**+2**	**+3**	**+4**
*Temp.* °C	>41	39–40.9	-	38.5–38.9	36–38.4	34–35.9	32–33.9	30–31.9	<39.9
*Heart rate*	>180	140–179	110–139	-	70–109	-	55–69	40–54	<39
*Respiratory rate*	>50	35–49	-	25–34	12–24	10–11	6–9	-	<5
*Serum sodium* mmol/L	>180	160–179	155–159	150–154	130–149	-	120–129	111–119	<110
*Serum potassium* mmol/L	>7	6–6.9	-	5.5–5.9	3.5–5.4	3–3.4	2.5–2.9	-	<2.5
*Serum creatinine* mg/100 mL	>3.5	2–3.4	1.5–1.9	-	0.6–1.4	-	<0.6	-	-
*Hematocrit (HT)*	>60	-	50–59.9	46–49.9	30–45.9	-	20–29.9	-	<20
*Leukocytes total*/mm^3^ × 1000	>40	-	20–39.9	15–19.9	3–14.9	-	1–2.9	-	<1
*Serum bicarbonate* mmol/L	>52	41–51.9	-	32–40.9	22–31.9	-	18–21.9	15–17.9	<15
*Dissemination score*	- Fournier’s gangrene confined to the urogenital and/or anorectal region, add “1” - Fournier’s gangrene confined to the pelvic region, add “2” - Fournier’s gangrene extending beyond the pelvic region, add “6”								
*Age score*	- Age ≥ 60 years, add “1” - Age < 60 years, add “0”								
**SFGSI**	High				Normal	Low			
*Serum potassium* mmol/L	>7	6–6.9	-	5.5–5.9	3.5–5.4	3–3.4	2.5–2.9	-	<2.5
*Serum creatinine* mg/100 mL	>3.5	2–3.4	1.5–1.9	-	0.6–1.4	-	<0.6	-	-
*Hematocrit (HT)*	>60	-	50–59.9	46–49.9	30–45.9	-	20–29.9	-	<20

**Table 2:** Main characteristics of the included studies.

Study, Year	Study Design	Single/Multi-Center	Sample, N	Gender, M/F	Mortality, %	Age, (Years) Mean ± SD/ Median (IQR)	Survivors’ Age, (Years) Mean ± SD/ Median (IQR)	Non-Survivors’ Age, (Years) Mean ± SD/ Median (IQR)
Yeniyol et al., 2004 [11]	Retrospective	Single-center	25	25/0	24.0%	61.7 ± 13.4	58.9 ± 12.5	70.6 ± 13.4
Tuncel et al., 2006 [12]	Retrospective	Single-center	20	20/0	30.0%	N.R.	60.0 ± 12.9	64.5 ± 6.5
Cocoran et al., 2008 [13]	Retrospective	Single-center	68	54/14	10.3%	55.8 ± 15.2	59.3 ± 11.8	55.4 ± 15.6
Kabay et al., 2008 [14]	Retrospective	Multi-center	72	67/5	40.3%	61 (24–87)	61 (27–87)	62 (42–87)
Unalp et al., 2008 [15]	Retrospective	Single-center	68	59/9	10.3%	54.7 ± 15.6	53.3 ± 15.8	66.7 ± 5.6
Luján Marco et al., 2009 [16]	Retrospective	Single-center	51	48/3	15.7%	63 (17–85)	60 (17–81)	73.5 (50–85)
Ulug et al., 2009 [17]	Retrospective	Single-center	20	20/7	18.5%	N.R.	53.9 ± 21.5	57.2 ± 12.9
Yilmazlar et al., 2010 [6]	Retrospective	Single-center	80	57/23	21.2%	57 (24–85)	55 (24–85)	62 (47–77)
Altarac et al., 2012 [18]	Retrospective	Single-center	41	39/2	36.6%	59 (51–69)	58 (47–66)	69 (45–78)
Aridogan et al., 2012 [19]	Retrospective	Single-center	71	71/0	29.6%	61.3 ± 12.3	61.2 ± 12.1	66.2 ± 12.4
Roghmann et al., 2012 [20]	Retrospective	Single-center	44	44/0	29.5%	59 (48–65)	52 (43–64)	62 (52–71)
Verma et al., 2012 [21]	Retrospective	Multi-center	95	81/14	27.4%	46.5 ± 15.6	N.R.	N.R.
Wang et al., 2012 [22]	Retrospective	Single-center	24	20/4	20.8%	N.R.	48.9 ± 12.9	46.6 ± 14.1
Sarvestani et al., 2013 [23]	Retrospective	Single-center	28	28/0	35.7%	44.6 ± 8.5	39.4 ± 8.9	54.1 ± 7.8
Vyas et al., 2013 [24]	Prospective	Single-center	30	30/0	20.0%	N.R.	35.7 ± 9.4	55.0 ± 9.5
Lin et al., 2014 [7]	Retrospective	Single-center	65	85/0	18.8%	N.R.	57.8 ± 14.4	62.3 ± 13.0
Oymaci et al., 2014 [25]	Retrospective	Single-center	16	10/6	18.7%	61.2 ± 12.3	61.7± 12.7	59.0 ± 12.1
Tuncel et al., 2014 [26]	Retrospective	Single-center	50	50/0	14.0%	61 (35–79)	58 (35–79)	68.5 (58–77)
Erol et al., 2015 [27]	Retrospective	Multi-center	99	99/0	17.2%	N.R.	60.9 ± 13.1	68.1 ± 12.6
Oguz et al., 2015 [28]	Retrospective	Single-center	43	34/9	13.9%	53.3 ± 16.1	50.1 ± 14.1	63.0 ± 18.6
Trachouli et al., 2015 [29]	Retrospective	Single-center	72	64/8	16.7%	51 (23–75)	N.R.	N.R.
Doluoğlu et al., 2016 [30]	Retrospective	Single-center	39	N.R.	20.5%	N.R.	65 (43–83)	52 (30–90)
Yim et al., 2016 [31]	Retrospective	Single-center	62	61/1	41.9%	N.R.	57.1 ± 14.4	56.2 ± 13.0
Üreyen et al., 2017 [32]	Retrospective	Multi-center	29	18/11	20.7%	51.5 ± 13.4	51.9 ± 13.5	50.0 ± 13.9
Elsaket et al., 2018 [33]	Retrospective	Single-center	44	43/1	11.4%	51 (28–82)	N.R.	N.R.
Pehlivanli et al., 2018 [34]	Retrospective	Single-center	23	19/4	21.7%	65.9 ± 16.3	63.0 ± 16.3	78.0 ± 10.8
Tenório et al., 2018 [35]	Retrospective	Single-center	124	N.R.	25.8%	50.8 ± 19.5	48.1 ± 18.6	58.5 ± 20.1
Watterauer et al., 2018 [36]	Retrospective	Single-center	20	20/0	15.0%	66 (46–73)	64 (43–72)	84 (67–94)
Arora et al., 2019 [37]	Prospective	Single-center	50	50/0	24.0%	53 ± 16.8	47.9 ± 14.4	69.9 ± 11.1
Citigez et al., 2019 [38]	Retrospective	Single-center	48	48/0	14.6%	53.9 ± 12.6	50.5 ± 9.8	73.4 ± 8.6
Karaali et al., 2019 [39]	Retrospective	Single-center	89	58/31	22.5%	60.2 ± 12.7	53.9 ± 13.6	67.6 ± 11.5
Egin et al., 2020 [40]	Retrospective	Single-center	30	16/14	26.7%	58.7 ± 11.5	55.1 ± 9.17	68.5 ± 12.1
Noegroho et al., 2021 [41]	Retrospective	Single-center	83	N.R.	27.7%	N.R.	45.9 ± 11.8	55.9 ± 13.6
Usta et al., 2021 [42]	Retrospective	Single-center	60	45/15	16.7%	61.4 ± 16.0	59.9 ± 16.1	68.5 ± 14.1
Çomçalı et al., 2022 [43]	Retrospective	Single-center	144	101/43	7.6%	55 (19–80)	61 (38–80)	53 (19–78)
Tosun et al., 2022 [44]	Retrospective	Single-center	150	123/27	15.3%	57.9 ± 13.2	56.0 ± 12.8	68.5 ± 10.4
Bozkurt et al., 2023 [45]	Retrospective	Single-center	28	28/0	39.3%	62.0 ± 18.7	54.5 ± 17.3	73.4 ±15.2
Egin et al., 2023 [46]	Retrospective	Single-center	73	42/31	23.3%	57.3 ± 13.4	69.2 ± 13.3	53.7 ± 11.2
Eray et al., 2023 [47]	Retrospective	Single-center	52	30/22	26.9%	54.3 ± 13.4	52.9 ± 13.6	57.9 ± 12.8
Ongaro et al., 2023 [48]	Retrospective	Single-center	20	20/0	35.0%	58 (51–88)	N.R.	N.R.

SD: Standard Deviation; IQR: Interquartile Range; N.R: Not Reported.

## Data Availability

Data presented are contained within the article; for additional information, datasets are also available upon request from the corresponding author.

## Supplementary Materials

The following supporting information can be downloaded at: https://www.mdpi.com/article/10.3390/jpm13091283/s1, Figure S1: Publication bias analysis using the Funnel Plot including studies reporting FGSI.; Figure S2: Publication bias analysis using the Funnel Plot including studies reporting SFGSI.; Figure S3: Publication bias analysis using the Funnel Plot including studies reporting UFGSI.; Figure S4: Galbraith Plot including studies reporting FGSI.; Figure S5: Galbraith Plot including studies reporting SFGSI.; Figure S6: Galbraith Plot including studies reporting UFGSI.; Table S1: National Institutes of Health (NIH) quality assessment tool for the included studies.
