# Supplementary material for: The Value of Fournier’s Gangrene Scoring Systems on Admission to Predict Mortality: A Systematic Review and Meta-Analysis
Source: J Pers Med. 2023 Aug 22;13(9):1283. doi: 10.3390/jpm13091283 (PMC10532663; doi:10.3390/jpm13091283)

Supplementary Table S1. National Institutes of Health (NIH) quality assessment tool for the included studies.

Questions	1	2	3	4	5	6	7	8	9	10	11	12	13	14	
Altarac, 2012	Yes	No	NA	No	No	Yes	Yes	NA	Yes	NR	Yes	NR	NA	Yes	i
Aridogan, 2012	Yes	Yes	NA	Yes	No	Yes	Yes	NA	Yes	NR	Yes	NR	NA	Yes	i
Arora, 2019	Yes	Yes	NA	Yes	No	Yes	Yes	NA	Yes	NR	Yes	NR	NR	Yes	i
Bozkurt, 2023	Yes	Yes	NA	Yes	No	Yes	Yes	NA	Yes	NR	Yes	NR	NA	Yes	i
Citgetz, 2019	Yes	Yes	NA	Yes	Yes	Yes	Yes	NA	Yes	NR	Yes	NR	NA	No	i
Cocoran, 2008	Yes	Yes	NA	Yes	No	Yes	Yes	NA	Yes	NR	Yes	NR	NA	Yes	i
Çomçalı, 2020	Yes	Yes	NA	Yes	Yes	Yes	Yes	NA	Yes	NR	Yes	NR	NA	Yes	i
Doluoğlu, 2016	Yes	Yes	NA	Yes	No	Yes	Yes	NA	Yes	Yes	Yes	NR	NA	Yes	i
Egin 2020	Yes	Yes	NA	No	No	Yes	Yes	NA	Yes	Yes	Yes	NR	NA	Yes	i
Eğin 2023	Yes	Yes	NA	No	No	Yes	Yes	NA	Yes	Yes	Yes	NR	NA	Yes	i
Elsaket, 2018	Yes	Yes	NA	Yes	Yes	Yes	Yes	NA	Yes	NR	Yes	NR	NA	No	i
Eray, 2021	Yes	Yes	NA	Yes	No	Yes	Yes	NA	Yes	NR	Yes	NR	NA	Yes	i
Erol, 2015	Yes	No	NA	No	No	Yes	Yes	NA	Yes	NR	Yes	NR	NA	Yes	i
Hong, 2017	Yes	Yes	NA	Yes	No	Yes	Yes	NA	Yes	NR	Yes	NR	NA	Yes	i
Kabay, 2008	Yes	Yes	NA	No	No	Yes	Yes	NA	Yes	Yes	Yes	NR	NA	Yes	i
Karaali, 2019	Yes	Yes	NA	Yes	No	Yes	Yes	NA	Yes	NR	Yes	NR	NA	Yes	i
Lin, 2014	Yes	Yes	NA	Yes	No	Yes	Yes	NA	Yes	NR	Yes	NR	NA	Yes	i
Luján Marco, 2009	Yes	Yes	NA	Yes	No	Yes	Yes	NA	Yes	NR	Yes	NR	NA	No	i
Noegroho, 2021	Yes	Yes	NA	Yes	No	Yes	Yes	NA	Yes	NR	Yes	NR	NA	Yes	i
Oguz, 2015	Yes	Yes	NA	Yes	No	Yes	Yes	NA	Yes	NR	Yes	NR	NA	Yes	i
Ongaro, 2023	Yes	Yes	NA	Yes	No	Yes	Yes	NA	Yes	NR	Yes	NR	No	Yes	i
Oymaci, 2014	Yes	Yes	NA	Yes	No	Yes	Yes	NA	Yes	NR	Yes	NR	NA	No	i
Pehlivanlı, 2018	Yes	Yes	NA	Yes	No	Yes	Yes	NA	Yes	NR	Yes	NR	NA	Yes	i
Roghmann, 2012	Yes	Yes	NA	Yes	No	Yes	Yes	NA	Yes	NR	Yes	NR	NA	Yes	i
Sarvestani, 2013	Yes	Yes	NA	Yes	No	Yes	Yes	NA	Yes	NR	Yes	NR	NA	YES	i
Tarchouli, 2015	Yes	Yes	NA	Yes	No	Yes	Yes	NA	Yes	NR	Yes	NR	NA	Yes	i
Tenório, 2018	Yes	Yes	NA	Yes	No	Yes	Yes	NA	Yes	NR	Yes	NR	NA	No	i
Tosun, 2022	Yes	Yes	NA	Yes	No	Yes	Yes	NA	Yes	NR	Yes	NR	NA	Yes	i
Tuncel, 2014	Yes	Yes	NA	Yes	No	Yes	Yes	NA	Yes	Yes	Yes	NR	NA	Yes	i
Ulug, 2009	Yes	Yes	NA	Yes	No	Yes	Yes	NA	Yes	NR	Yes	NR	NA	Yes	i
Unalp, 2008	Yes	Yes	NA	Yes	No	Yes	Yes	NA	Yes	NR	Yes	NR	NA	Yes	i
Ureyen, 2017	Yes	Yes	NA	No	No	Yes	Yes	NA	Yes	NR	Yes	NR	NA	Yes	i
Usta, 2020	Yes	Yes	NA	Yes	No	Yes	Yes	NA	Yes	NR	Yes	NR	NA	Yes	i
Verma, 2012	Yes	Yes	NA	No	No	Yes	Yes	NA	Yes	NR	Yes	NR	NA	Yes	i
Vyas, 2013	Yes	Yes	NA	Yes	No	Yes	Yes	NA	Yes	NR	Yes	NR	NA	Yes	i
Wang, 2012	Yes	Yes	NA	Yes	No	Yes	Yes	NA	Yes	NR	Yes	NR	NA	Yes	i
Wetterauer, 2018	Yes	Yes	NA	Yes	No	Yes	Yes	NA	Yes	NR	Yes	NR	NA	Yes	i
Yeniyol, 2004	Yes	Yes	NA	Yes	No	Yes	Yes	NA	Yes	Yes	Yes	NR	NA	Yes	i
Yilmazalar, 2010	Yes	Yes	NA	Yes	No	Yes	Yes	NA	Yes	NR	Yes	NR	NA	No	i
Yim, 2016	Yes	Yes	NA	Yes	No	Yes	Yes	NA	Yes	NR	Yes	NR	NA	Yes	i

Quality was rated as 0 for poor (0–4 out of 14 questions), i for fair (5–10 out of 14 questions), or ii for good (11–14 out of 14 questions); NA: not applicable, NR: not reported.

Supplementary Figure S1. Publication bias analysis using the Funnel Plot including studies reporting FGSI.

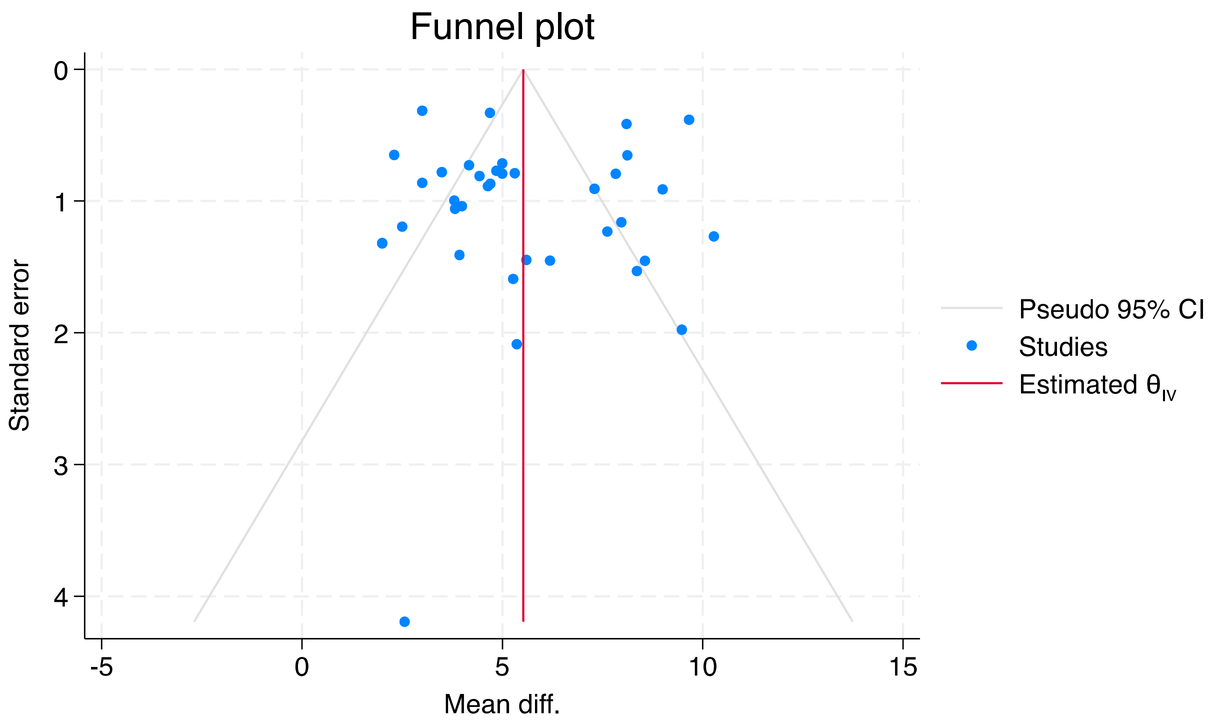

Supplementary Figure S2. Publication bias analysis using the Funnel Plot including studies reporting SFGSI.

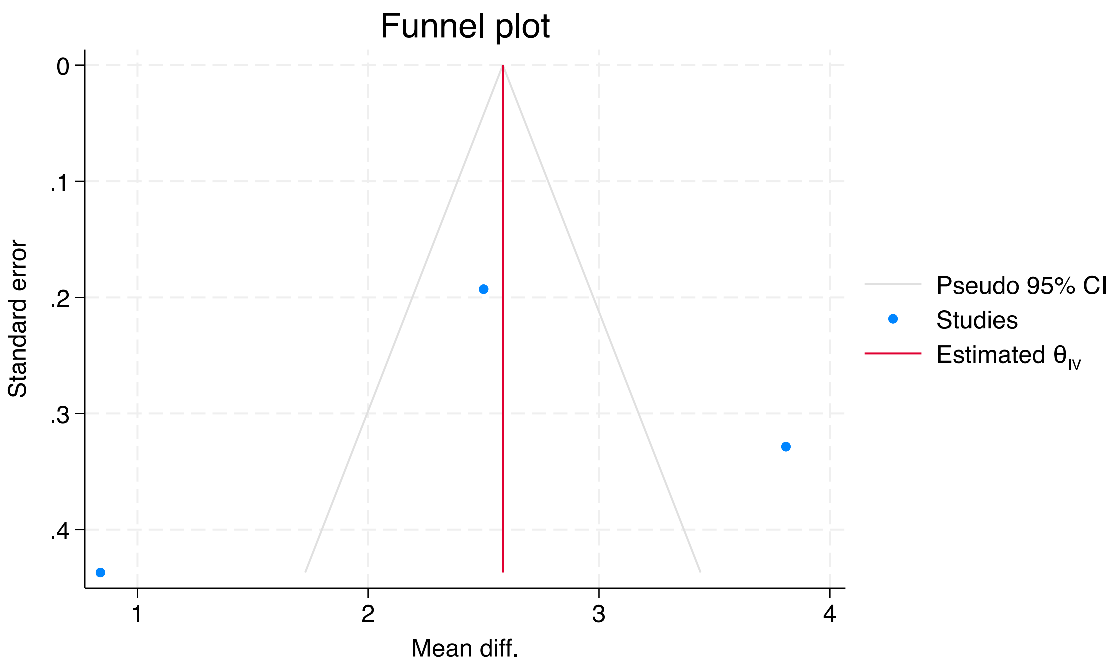

Supplementary Figure S3. Publication bias analysis using the Funnel Plot including studies reporting UFGSI.

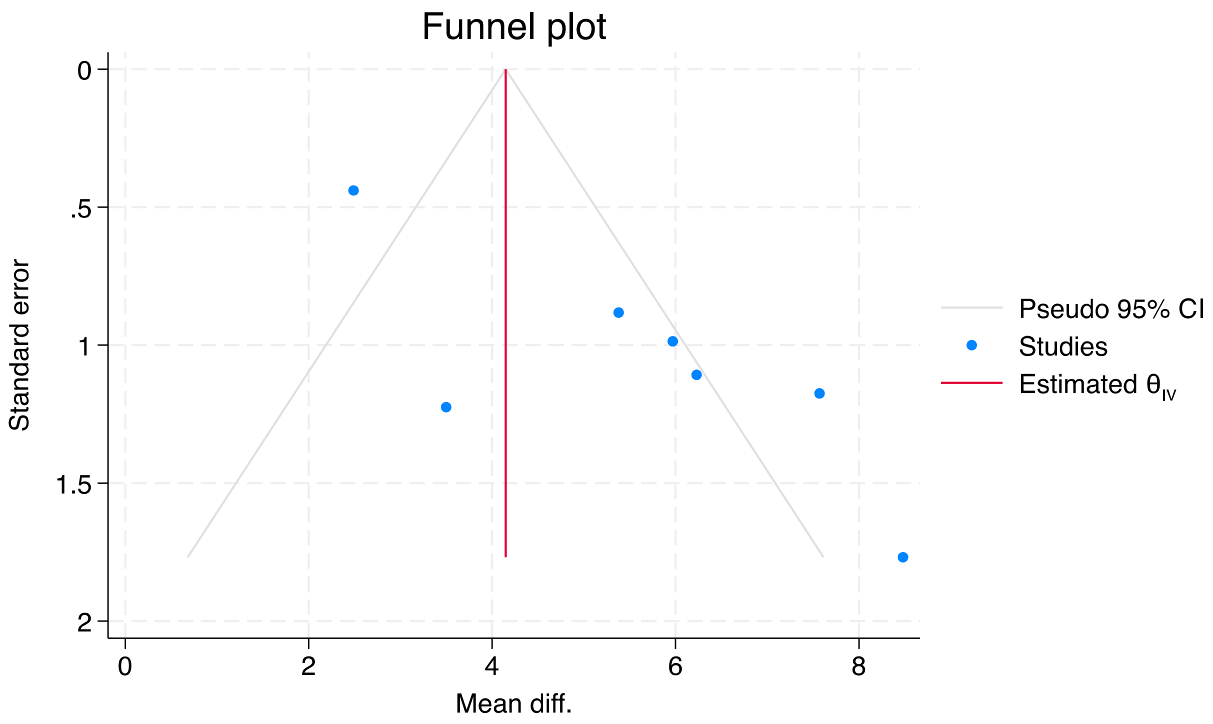

Supplementary Figure S4. Galbraith Plot including studies reporting FGSI.

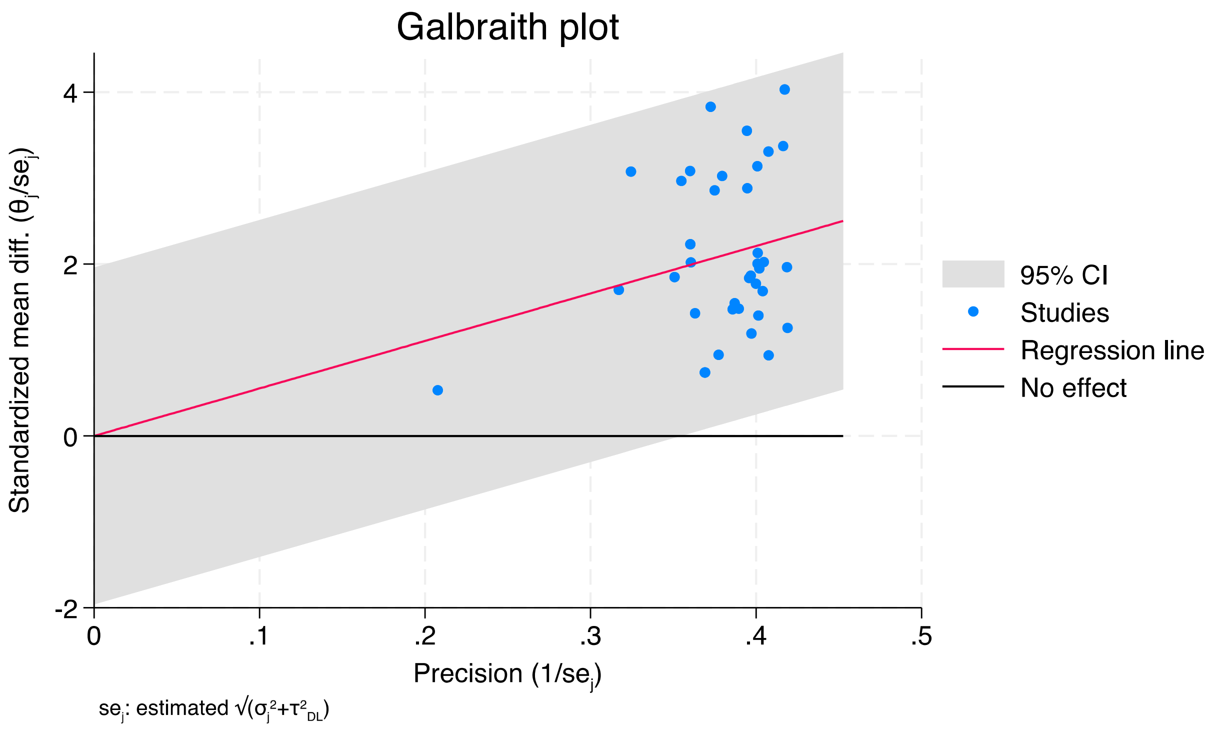

Supplementary Figure S5. Galbraith Plot including studies reporting SFGSI.

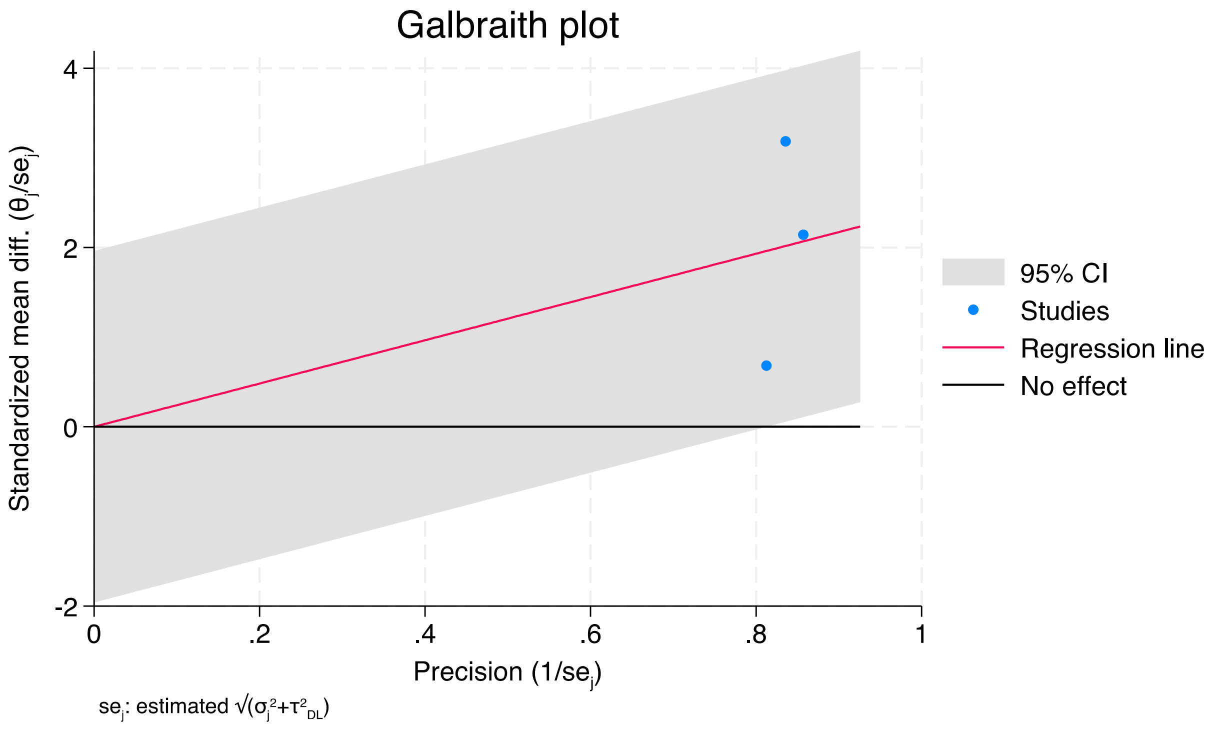

Supplementary Figure S6. Galbraith Plot including studies reporting UFGSI.

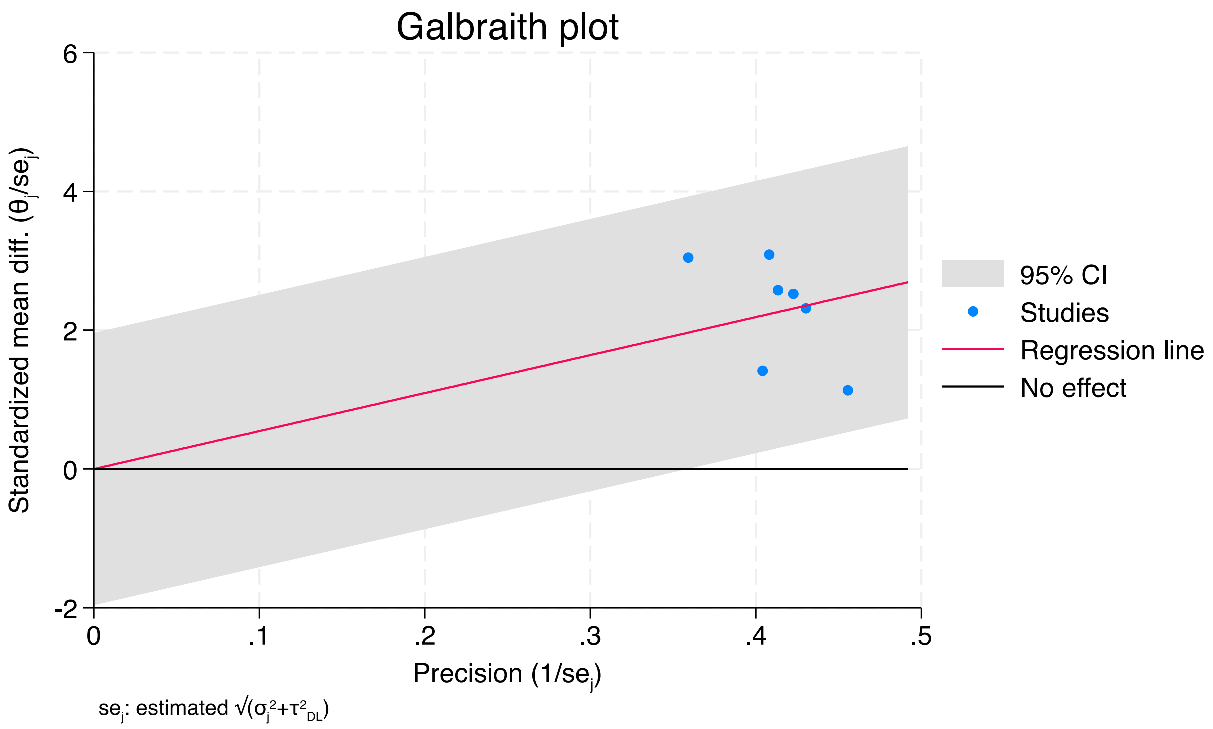

Supplement: Supplementary file 1 [file jpm-13-01283-s001.zip › jpm-2570627-supplementary.pdf]
